# Supplementary material for: Wnt/β-Catenin Signaling Regulates Yap/Taz Activity during Embryonic Development in Zebrafish
Source: Int J Mol Sci. 2024 Sep 17;25(18):10005. doi: 10.3390/ijms251810005 (PMC11432159; doi:10.3390/ijms251810005)
Supplement: Supplementary file 1 [file ijms-25-10005-s001.zip › ijms-3061375-supplementary.pdf]

A

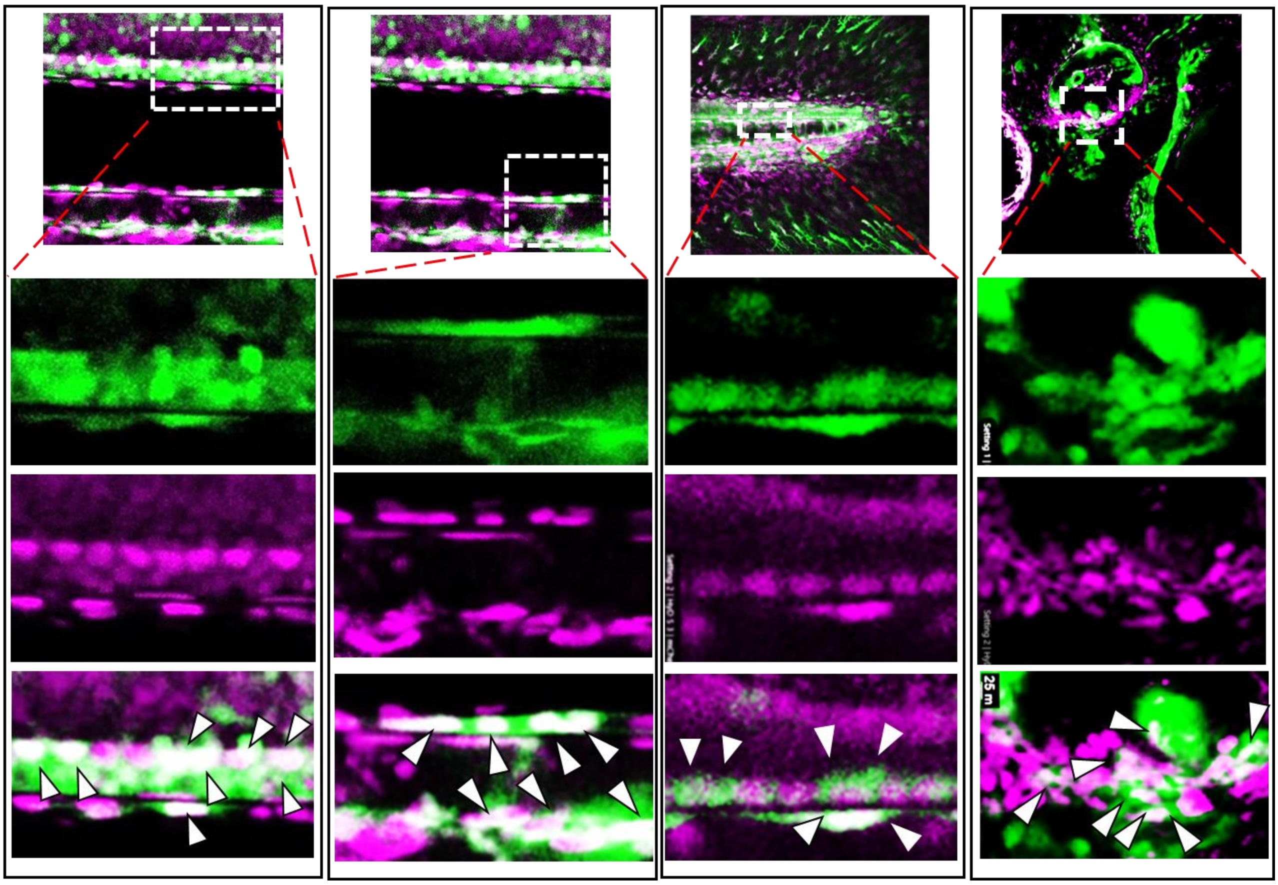

B

Image Calculator (Process >Image Calculator) was used to generate the intersection (AND operator) between the 2 signals

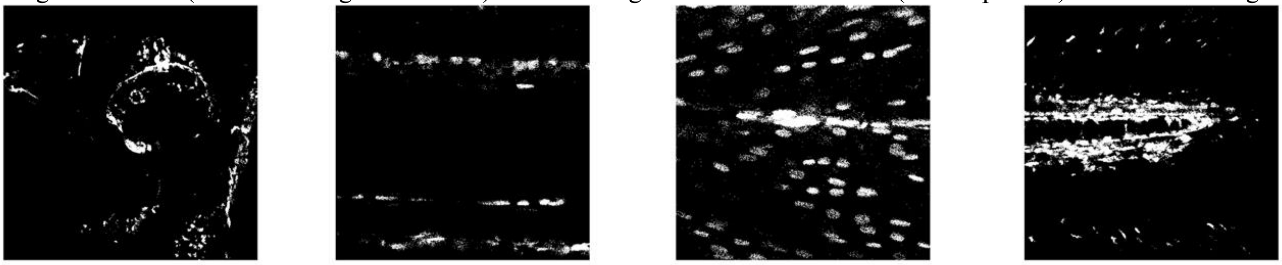

Figure S1: (**A**) Representative merged images of 48 hpf Tg(7xTCF-Xla.Siam:GFP)[ia4] and Tg(Hsa.CTGF:nlsmCherry)[ia49] embryos showing Wnt (green) and Yap/Taz (magenta) responsive areas (upper raw). Magnification of specific areas of different districts of the embryos show the presence of cells with a co-expression in single 2D scans. Arrowheads indicate the presence of partial co-localization between the mCherry and GFP signals in different regions of the embryo (**B**) In ImageJ, the 'Image Calculator' tool (accessible via *Process > Image Calculator*) was utilized to compute the intersection of two signal images using the 'AND' operator. This process combines the two images by retaining only the areas where both images have overlapping or matching features, effectively highlighting common regions between the two signals.

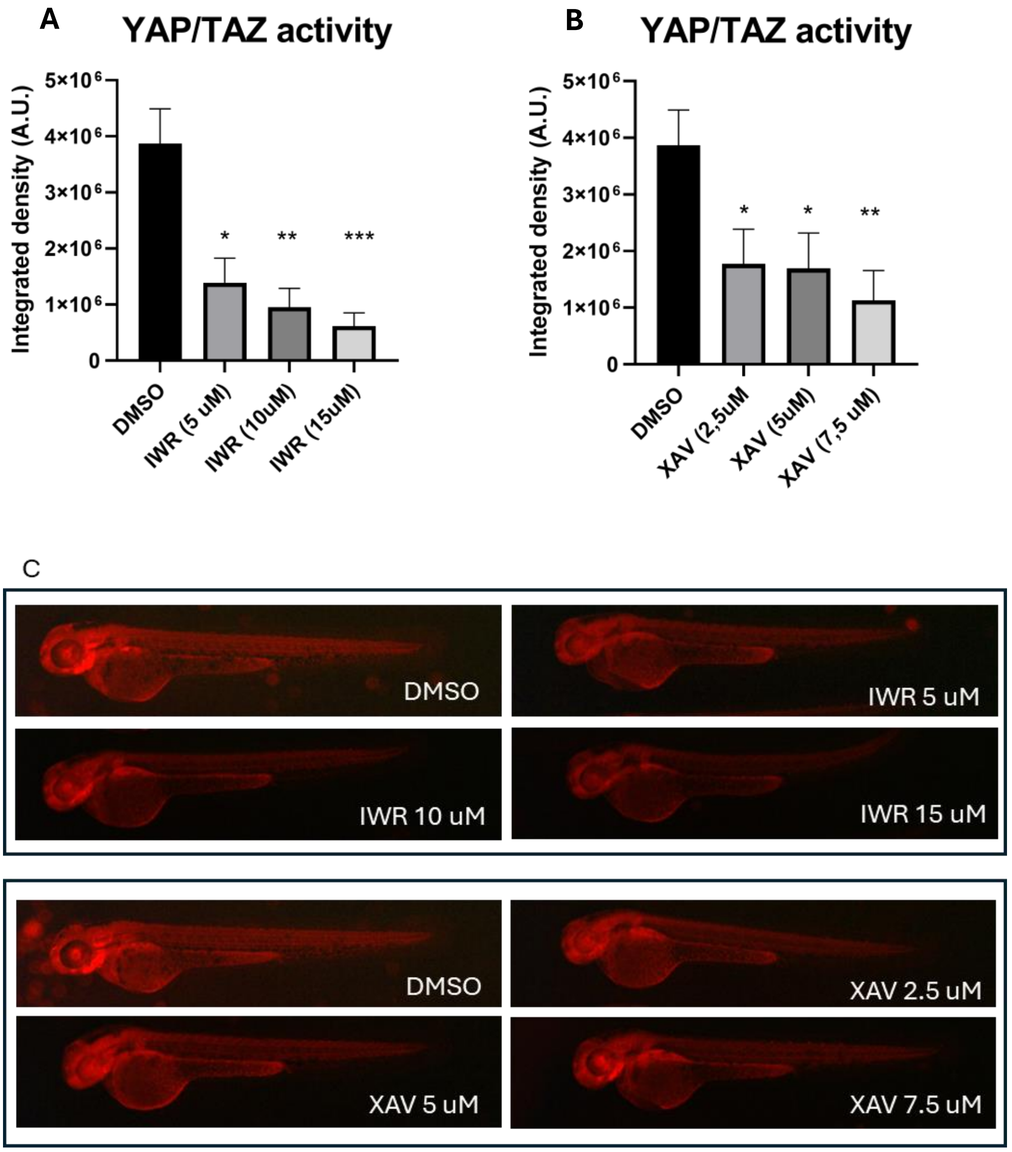

Figure S2: YAP/TAZ Tg(Hsa.CTGF:nlsmCherry)^{ia49} reporter embryos (C) were exposed to IWR-1 or XAV939 for 24 hours, and the fluorescent reporter expression was imaged and quantified (A and B) at the level of the whole embryo at 48 hpf (C). mCherry fluorescence levels were analysed by measuring the integrated density with FIJI software. Data are presented as mean +/- SEM (Mann-Whitney test (B), *p<0.05, **p<0.01, ***p<0.001.

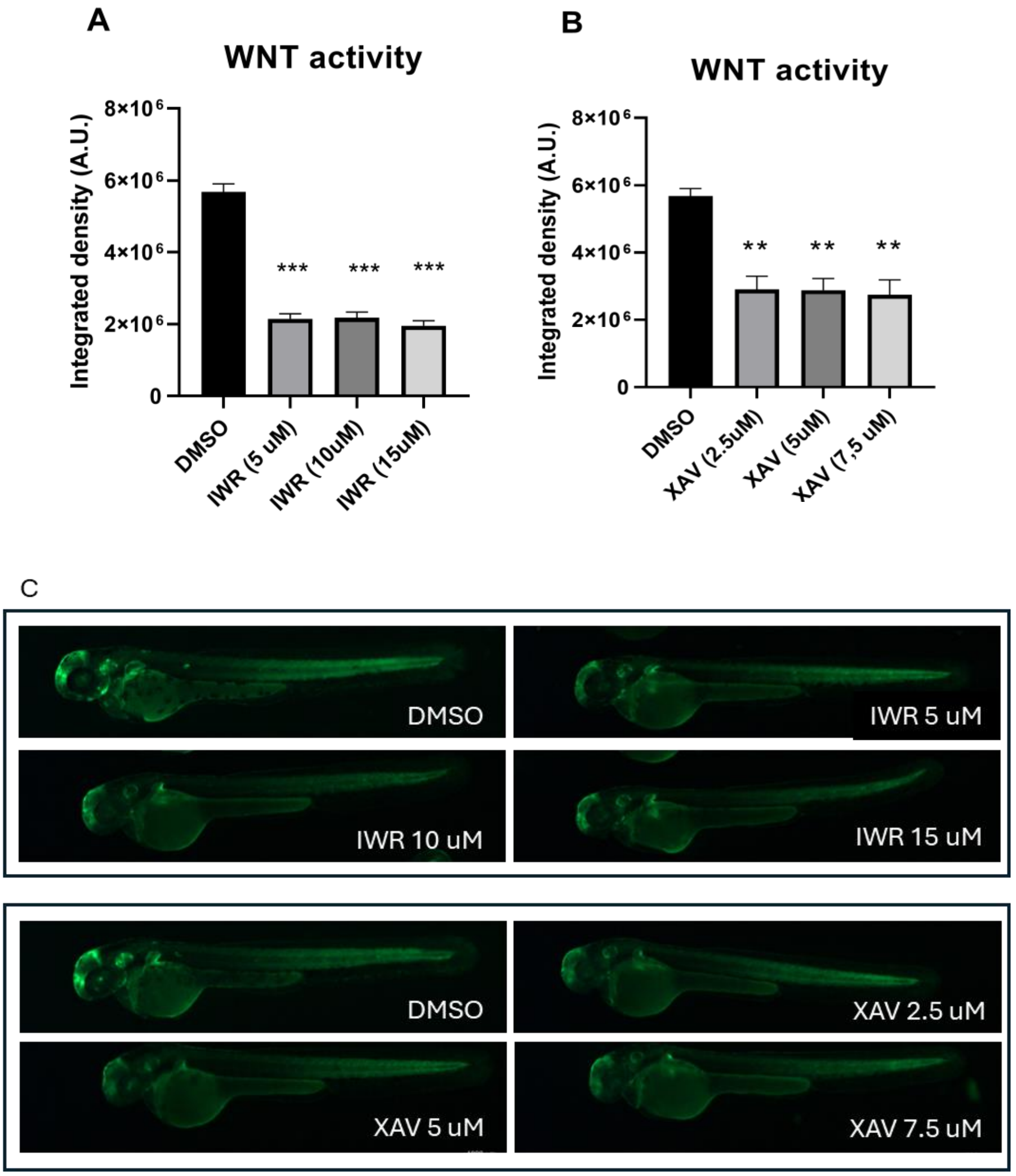

Figure S3: Wnt Tg(7xTCF-Xla.Siam:GFP)^{ia4} zebrafish reporter embryos (C) were exposed to IWR-1 or XAV939 for 24 hours, and the fluorescent reporter expression was quantified (A and B) at the level of the whole embryo at 48 hpf (C). eGFP fluorescence levels were analysed by measuring the integrated density with FIJI software. Data are presented as mean +/- SEM (Mann-Whitney test (B); **p<0.01, ***p<0.001.
